# Tailored Multibody Tibiofemoral Joint Model for Precision Care

**DOI:** 10.1155/abb/5951085

**Published:** 2025-11-10

**Authors:** Shavkat Nadir Kuchimov, Mehmed Ozkan, Yener Temelli

**Affiliations:** ^1^ Institute of Biomedical Engineering, Bogazici University, Istanbul, Türkiye, boun.edu.tr; ^2^ Motion Analysis Center, Istanbul Kultur University, Istanbul, Türkiye, iku.edu.tr; ^3^ Department of Orthopedics, American Outpatient Medical Center, American Hospital, Istanbul, Türkiye, amerikanhastanesi.org

**Keywords:** knee contact surfaces, knee joint model, patient specific ligament attachments, patient specific ligament force modeling, patient specific model, surgical planning, tibiofemoral joint model

## Abstract

Knee motion involves intricate coordination among various anatomical structures. Effective treatment of knee pathologies requires precise identification of deformities and accurate surgical interventions, which often involve rapid tissue modification based on established knowledge. However, motion disorders are typically detected long after surgery. To address this, a simulation environment is proposed to plan and analyze surgical impacts on knee motion. Comprehensive knee joint modeling is crucial for a successful simulation. Clinically accepted movement procedures based on passive knee motion make tibiofemoral articulation modeling sufficient. Proposed model tibiofemoral articulation, incorporating 15 ligaments, tibial and femoral bones, and cartilages. Ligaments’ tensile, bones’, and cartilages’ contact forces (CFs) define internal force interactions. Anatomical structures, their shapes, positions, and attachment points are identified from MRI, ensuring patient‐specific modeling. Simulation results are compared to cadaver data using passive knee motion. Two rotational and three translational dependent joint motions (JMs) are compared pairwise. The results are highly correlated with the clinical benchmark. Pearson’s correlation show a strong association between experimental and simulated passive knee flexions (PKFs; *r* > 0.89). The comparison is statistically significant with *p* < 0.05. Anterior–posterior translation showed the highest correlation (*R* ^2^ = 0.994). The findings indicate that the simulated model closely replicates actual knee responses.

## 1. Introduction

The human knee joint is a complex structure bearing the whole body during various activities resulting in huge local force and torque generations. Hence, it is one of the most frequently injured joints. An in‐depth understanding of joint dynamics helps to comprehend injury mechanics, assessment of tissue loading, and consequences of surgical intervention. The sound knee joint has a nonhomogeneous oval shape and smooth surface at the distal end of the femur consisting of medial and lateral condyles. It has a varying curvature radius of instantaneous contact points at the sagittal plane and posterior cross sections as illustrated in Figure 1a,b. In the sagittal plane, hypothetical contact circles have a lower radius at higher flexion angles than near full extension angles [1, 2]. In the frontal plane, the contact surface of the medial condyle is a circular arc and the lateral condyle surface is flattened at full extension (Figure 1c) [3]. Posterior cross sections of condyles are circular as shown in Figure 1d and their radii are not uniform for the range of the knee flexion motion. Femoral condyles are mechanically coupled with the proximal head of the tibia containing plateaus which have nonhomogeneous medial concave and lateral saddle shapes [4, 5]. Contact of these surfaces, made of bones and cartilage, produces contact forces (CFs).

Figure 1Cross sections of the distal femur depict femoral condyle contact surfaces. Sagittal sections of the medial (a) and lateral (b) condyles, segmented from MRI, show varying radii of curvatures, adapted from Smith et al. [1] and Kapandji [2]. The medial condyle’s frontal section (c) forms a circular arc, while the lateral condyle appears flat in full extension. Transverse sections (d) reveal posterior circular shapes of both condyles during higher knee flexion, adapted from Freeman and Pinskerova [3].

**Figure:**
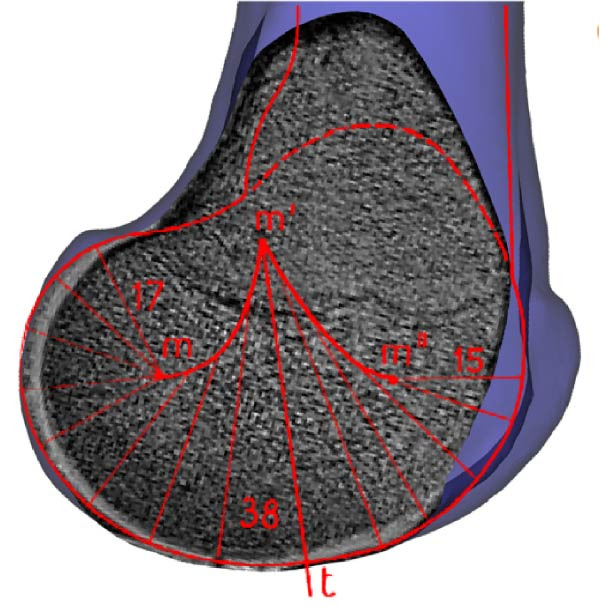
(a)

**Figure:**
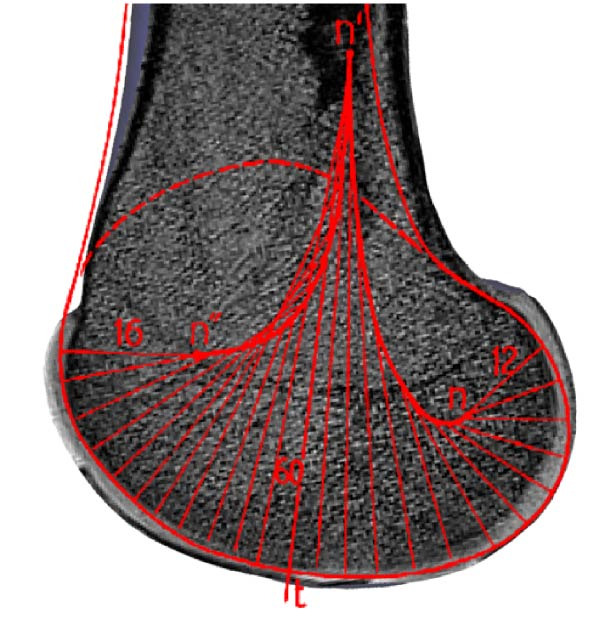
(b)

**Figure:**
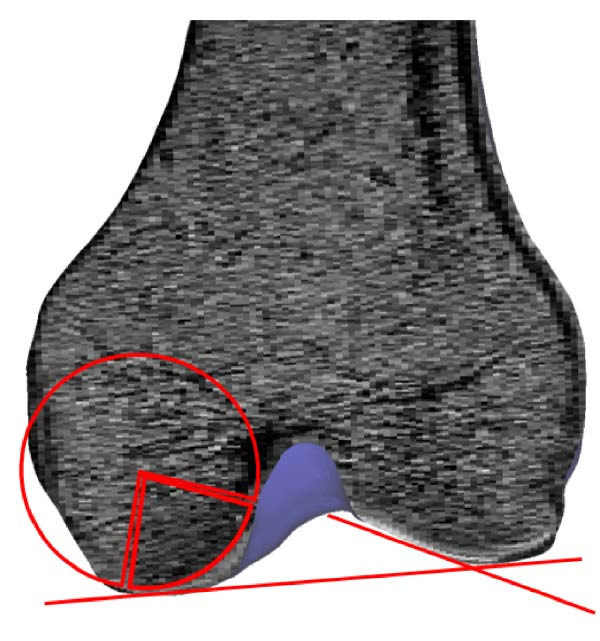
(c)

**Figure:**
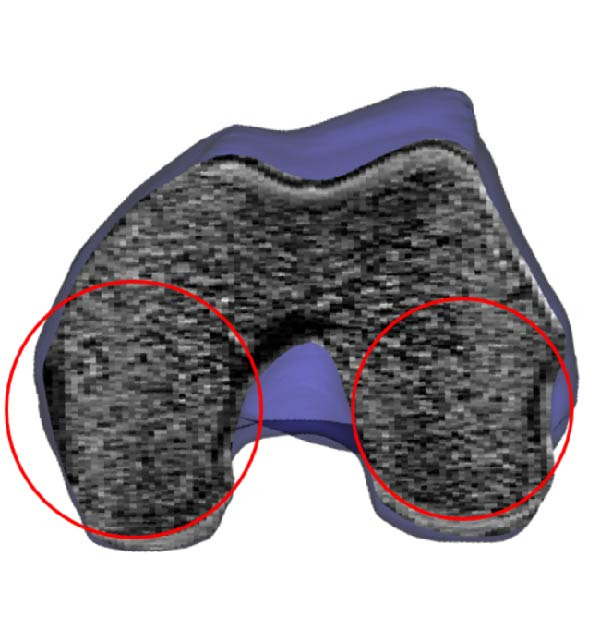
(d)

Anatomically, ligaments and capsule have attachment sites on the femur, tibia, and fibula. Under tension, these ligamentous structures become a source of passive ligament forces (LFs). The interaction of CF and LF together defines the main force structure. Therefore, a generic comprehensive knee model must possess all these force components customized to a subject.

The literature studies generally focuses on the CF related to the geometry of anatomical surfaces, and the constraint forces associated with connective tissue. The anatomical representation varies depending on the level of details considered for the knee joint. Several computational knee models employ anthropometric parameters. For instance, Abdel‐Rahman and Hefzy [6] and Wilson et al. [7] approximate the femoral condyles with a pair of spherical shells. They fitted spheres to the medial and lateral condyles and used the radii as an anatomical feature. They and Shelburne et al. [8] treated medial and lateral tibial plateau surfaces as inclined planes, with slopes measured from specimens [6–8]. Although femoral condyle diameters and tibial plateau slopes contribute to anatomical accuracy, articular surfaces do not have a uniform condylar radii nor do plateau slopes completely define contact surfaces. Later knee morphology studies showed that femoral condyles have varying radii and tibial plateaus are not necessarily planar [1, 3].

In a study by Akalan et al. [9], the tibia with flat surfaces constrained by isometric ligaments was moved on the fixed femur (FF), resulting in contact traces resembling anatomical condyle contours. However, these curves do not represent the entire surface morphology of the condyles, thus, limiting the model’s ability to calculate internal–external, abduction–adduction rotations, and lateral–medial translation motions that are out of the contact pattern.

Some studies employ scanned or probed surface geometries of specimen knee articulation, contour or surface functions are derived in a way to simplify the calculations of geometric and dynamic equations. In Wismans et al.’s [10] model, generalized surfaces of femoral and tibial condyles are approximated by higher‐order mathematical polynomials. However abduction–adduction knee rotation cannot be studied due to assumptions of persisting permanent contacts on medial and lateral condyles [10].

Gerus et al. [11] showed that subject‐specific tibiofemoral joint geometry improves CF prediction on the medial side compared to generic models. Recent studies utilize radiology imaging modalities to extract contact surfaces at the knee joint [12–15]. Multibody computational models (CMs) presented by Guess et al. [15] based on cadaver and subject studies, consider the main interacting force components including menisci. However, knee capsules are often excluded from models.

In this study, we propose a multibody anatomic tibiofemoral model. Unlike existing approaches, we introduce a more generic model that covers a range of knee motions. With its comprehensive connective tissue and 3D contact surfaces, the proposed tibiofemoral model facilitates patient specificity, resulting in a simulation of the six degrees of freedom (DoF) JM during passive knee flexion (PKF).

## 2. Method

We construct a tibiofemoral compartment of the knee using a set of mechanical components. In the proposed model, these components can take the form of either rigid bodies or connective elements. Rigid bodies represent bones or cartilages, while contact surfaces and attachment sites for connective tissue are defined in relation to these rigid bodies. The shapes and sizes of the rigid bodies, as well as the ligament attachment sites are tailored to patient.

### 2.1. Rigid Body Coordinate System (CS) and Joint Axis (JA)

A knee joint involves four main bones as rigid body structures: the femur, tibia, patella, and fibula. It is common to treat the tibia and fibula as one rigid body. We exclude the patella from consideration due to its insignificant effect on PKF. The rigid body coordinate frames and JAs are constructed as described by Grood and Suntay [16], The femoral and tibial cartilages are attached to the distal head of the femur and the proximal head of the tibia.

#### 2.1.1. Cartesian CS of Rigid Bodies

**Figure 2:**
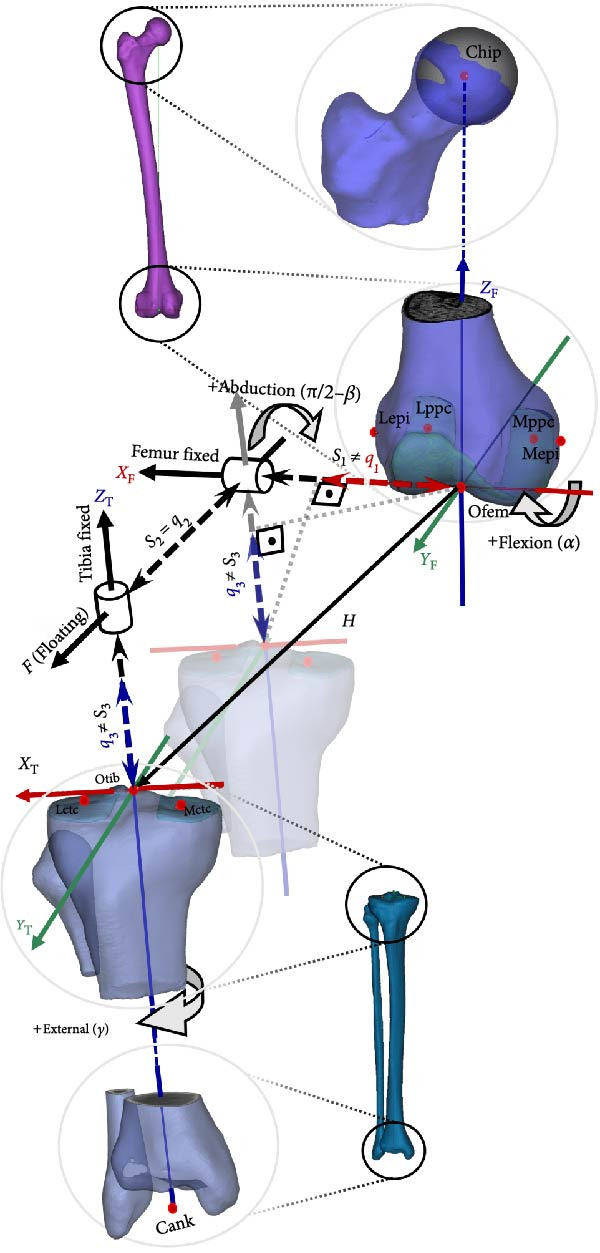
Femoral and tibial CSs are defined using anatomical landmarks as red dots, with axes in red (*X*), green (*Y*), and blue (*Z*), labeled with subscript F (femur) and T (tibia). Thick black arrow lines indicate joint coordinate axes for rotations: flexion–extension (*α*) about *X*_F_, tibial internal–external rotation (*γ*) about *Z*_T_, and abduction–adduction (π/2 − *β*) about a floating axis (*F*). The translation vector (*H*), shown as thin arrow line, comprised of lateral–medial (*S*_1_), anterior–posterior (*S*_2_, equal to clinical drawer *q*_2_), and superior–inferior (*S*_3_) components illustrated as dashed thick arrow lines. In abducted/adducted joints, *S*_1_ and *S*_3_ differ from clinical thrust (*q*_1_) and distraction–compression (*q*_3_), which are *H* projections on fixed axes, illustrated on a transparent tibia moving along *F*.

Rigid body coordinate frames are defined at the distal femoral and proximal tibial heads using anatomical landmarks illustrated as red dots in Figure 2. Femoral landmarks include the lateral (Lepi) and medial (Mepi) epicondyles, the most posterior points on the medial (Mppc) and lateral (Lppc) condyles, the hip ball center (Chip) and femoral origin (Ofem) which is the most distal point on the posterior surface of the femur midway between Lepi and Mepi. The vector pointing from Ofem to Chip is the longitudinal axis of the femur, *Z*_F_. *Y*_F_ is the cross product of *Z*_F_ with the unit vector parallel to the vector pointing from Mppc to Lppc. Finally, *X*_F_ is the cross product of *Z*_F_ and *Y*_F_ base vectors.

The tibia has four anatomical landmarks as seen in Figure 2. Two of them are the center points of the medial (Mctc) and lateral tibial condyles (Lctc). The ankle center (Cank) is the midpoint between the medial and lateral malleolus at the ankle joint. The origin of the tibial CS (Otib) is the midpoint between the tibial eminences. The tibial mechanical axis is a vector pointing from Cank to Otib, referred to as the *Z*_T_‐axis. *Y*_T_ is obtained by the cross product of *Z*_T_ and the unit vector directed from the point Mctc to Lctc. The third axis, *X*_T_, is orthogonal to *Z*_T_ and *Y*_T_.

#### 2.1.2. JA

Three base axes of the joint CS are depicted as black arrows in Figure 2. The two JAs are embedded in the femur and tibia, coinciding with the femoral *X*_F_ and tibial *Z*_T_ axes, respectively. The third JA, *F*, is floating and always orthogonal to both *X*_F_ and *Z*_T_. Rotational motions—flexion–extension (*α*), external–internal (*γ*), and abduction–adduction (π/2 − *β*)—occur about the *X*_F_, *Z*_T_, and *F* axes, respectively.

The combination of joint translation (JT) is represented by the vector *H*, pointing from the femoral to the tibial coordinate origin. The components of *H* along joint coordinate axes are JTs (indicated by dashed black lines with arrowheads), while projections of *H* onto JAs are clinical translations (represented by dashed colored lines with arrowheads). Although JT (*S*_2_) always equals clinical drawer (*q*_2_) along the floating axis (*F*), this is not the case for translations along the fixed axis. Lateral–medial (*S*_1_) and superior–inferior (*S*_3_) JTs are not equal to clinical lateral–medial trust (*q*_1_) and clinical distraction–compression (*q*_3_). In Figure 2, the tibia is posteriorly moved to eliminate anterior–posterior translation for illustration purpose of joint and clinical translations. For both rigid bodies, the positive directions are proximally for the *Z*‐axis, anteriorly for the *Y*‐axis, laterally for the right limb’s *X*‐axis, and medially for the left limb’s *X*‐axis.

### 2.2. Data Prep and Preprocessing

#### 2.2.1. Specimen Repository

To validate the proposed knee model, we used a repository of natural knee (NK) data [13, 14]. MRI, landmark points, and trajectories of segment motions were used within the dataset. We transformed these computed trajectories into the CM’s frame of reference for comparison purposes. Therefore, the repository data serves as the gold standard to verify the results of the proposed model.

The proposed method achieves patient specificity by utilizing the MRI of the subject. Patient‐dependent bone and cartilage geometries are segmented from MRI, along with ligament and capsule attachment sites, which are also patient specific. Ligament and capsule attachment sites are located on the MRI based on previous anatomy studies [5, 17–32].

The identified landmarks and segmented structures provide the basis for creating a twin model of the cadaver in the simulation environment. PKF motion data provided with the repository is used to benchmark the kinematics of our model.

#### 2.2.2. Segmentation and Landmark Identification

In this study, we utilize MIMICS to extract bone and cartilage segments and identify anatomical landmarks in 3D [33, 34]. We use segmented bone and cartilage surfaces for computing CFs. We define ligament attachment sites using anatomical landmarks on the bone structures.

**Figure 3:**
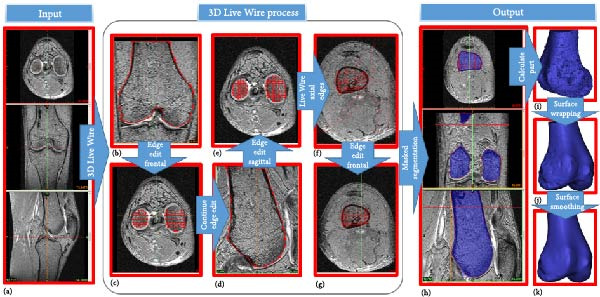
Extraction of femoral bone from MR images using Mimics. (a) Transverse, frontal, and sagittal MRI are imported. (b–d) 3D Live Wire detects bone surfaces from sample contours on coronal and sagittal planes, edited manually to remove spurious edges. (c–e) Contour traces appear as horizontal and vertical lines on axial images. (f) Axial contours are auto‐generated and (g) manually refined. (h) Created volume mask is illustrated for three orthogonal views. (i) A surface object is calculated. (j) The surface wrapping is applied to eliminate holes and bumps. (k) Final smoothing improves surface accuracy for dynamic contact force computation.

Initially, MRI are imported into MIMICS to establish orientation in three orthogonal views as in Figure 3a. Subsequently, bone and cartilage masks are identified using the 3D Live Wire process interactively, as illustrated in Figure 3b–g. The process begins with user‐defined segmentation points, and contours between these points are automatically generated based on an energy minimization function. Contours defined in the coronal (Figure 3b) and sagittal (Figure 3d) planes appear as horizontal lines (Figure 3c) and vertical red lines (Figure 3e), respectively, in the axial view. From these contours, edges for bones and cartilage are automatically generated (Figure 3f), followed by manual editing to remove artifacts in the axial images (Figure 3g).

In the final stage of the 3D Live Wire process, bone and cartilage tissue are segmented in all slices and three orthogonal views, illustrated as blue masks in three subparts of Figure 3h. A 3D object is constructed using these masks (Figure 3i). Spurious holes are removed using Surface Wrap function of MIMICS (Figure 3j). Additionally, the 3D Smooth Function is applied to reduce spurious rough or sharp structures, resulting in a more realistic bone surface (Figure 3k).

Coordinate points for the anatomical landmarks, ligament, and capsule attachment sites are manually identified on the MRI within the MIMICS environment with respect to segmented bones. Locations are then cross‐referenced with existing literature for validation [5, 17–32].

#### 2.2.3. Transformation Into Simulation Environment

ADAMS utilized in this study to model the motion and forces of interconnected bodies [35]. Within ADAMS, we construct the proposed tibiofemoral model, wherein bones, cartilage, and ligaments constitute the interconnected components. The dynamics of the tibiofemoral model are defined by bone and cartilage CFs, as well as ligament strain. The surface vertices of the bones and cartilages, are transferred from MIMICS into ADAMS and aligned with the established coordinate frames.

Anatomical landmarks, attachment sites, and the initial position of segments based on MRI CSs are transformed into the JCS using MATLAB software [36]. Additionally, the experimental data are visually inspected against any spurious points. Data are parametrized with respect to flexion angle to establish a common dependency for kinematics.

### 2.3. CM

#### 2.3.1. Ligament Placement and Tension Modeling

**Figure 4:**
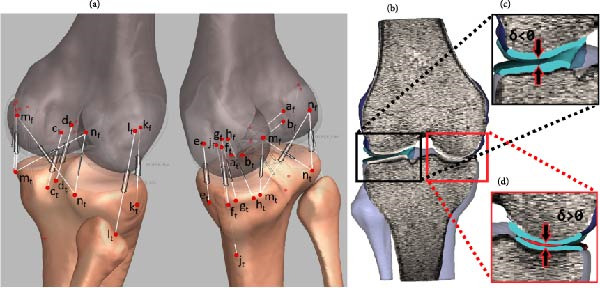
(a) Ligament insertion points are used to define the magnitude of current and reference vectors: aACL (*a*_t_, *a*_f_), pACL(*b*_t_, *b*_f_), aPCL(*c*_t_, *c*_f_), pPCL(*d*_t_, *d*_f_), dMCLa (*e*_t_, *e*_f_), dMCLp(*f*_t_, *f*_f_), oMCLa(*g*_t_, *g*_f_), MCLp(*h*_t_, *h*_f_), sMCL(*j*_t_, *j*_f_), ALL(*k*_t_, *k*_f_), LCL(*l*_t_, *l*_f_), OPL(*m*_t_,*n*_f_), APL(*n*_t_, *m*_f_), mCAP (*m*_t_, *m*_f_), and lCAP(*l*_t_, *l*_f_). (b) Penetration depth (*δ*) for cartilage is illustrated, (c) nonpenetration (*δ* > 0), and (d) penetration (*δ* < 0).

Figure 4a illustrates the attachment locations and line of action for all tensile elements corresponding to ligaments and capsules. A total of 15 tensile elements are defined. The insertion points are established with respect to coordinate points introduced in previous sections. Following the footsteps of earlier studies, cruciate ligaments are constructed as double bundles: an anterior bundle of the anterior cruciate ligament (ACL) denoted as aACL(*a*_t_, *a*_f_) and a posterior bundle denoted as pACL(*b*_t_, *b*_f_), similarly for the posterior cruciate ligament (PCL) an anterior bundle denoted as aPCL(*c*_t_, *c*_f_) and a posterior bundle denoted as pPCL(*d*_t_, *d*_f_) [17–19]. The oblique and deep medial collateral ligaments (oMCL and dMCL) in the medial compartment of the knee are also presented as pairs of force elements due to their wide nature. The oMCL has anterior bundle oMCLa(*g*_t_, *g*_f_) and posterior bundle oMCLp(*h*_t_, *h*_f_), similarly, the dMCL has anterior bundle dMCLa(*e*_t_, *e*_f_) and posterior bundle dMCLp(*f*_t_, *f*_f_) [20–22]. The remaining ligaments, including the superficial MCL (sMCL(*j*_t_, *j*_f_)), lateral collateral ligament (LCL(*l*_t_, *l*_f_)), anterior lateral ligament (ALL(*k*_t_, *k*_f_)), oblique popliteal pigament (OPL(*m*_t_, *n*_f_)), and arcuate popliteal ligament (APL(*n*_t_, *m*_f_)), are represented as single force elements, with attachments determined using MRI and anatomy studies [5, 20, 22–32]. Forces associated with the knee capsule, medial capsule (mCAP(*m*_t_, *m*_f_)), and lateral capsule (lCAP(*n*_t_, *n*_f_)) are included as described in previous studies [6, 21, 24, 37, 38].

The magnitudes of the ligament and capsule tensions are calculated using the force–elongation relationship as in Equation (1) [6, 7, 9, 10, 39–41]. The force acting on the insertion points (Figure 4a) along the line of sight carries tensile load only when the ligament length *L* exceeds the unstrained slack length *L*_0_. Due to intrinsic properties, ligamentous tension exhibits a “toe region” in the initial stages of ligament strain, followed by a linear force–length relation. The former nonlinear region has a quadratic relationship with the stiffness coefficient, denoted as *K*_*Q*_, while the latter is characterized by the stiffness parameter *K*_*L*_. The strain *ɛ* in the ligament is calculated using the instantaneous length *L* and slack length *L*_0_ as shown in Equation (2).

Conventionally, the strain threshold *ɛ*_1_ is assumed constant for all ligaments, typically set to 0.03. However, in this study, to ensure a smooth transition from the non‐linear to the linear region, *ɛ*_1_ is computed individually for each tensile element as in Equation (1).

(1) F=0ɛ<0KQL−L020≤ɛ≤ɛ1KLL−L01+ɛ1ɛ1<ɛ.

(2) ɛ=L−L0/L0.

In Equation (3), the slack length *L*_0_ for each ligament is computed using the expansion constant *e* and the ligament length when the knee is in full‐extension, *L*_*e*_.

(3) L0=eLe.

#### 2.3.2. Contact Surfaces and Forces

Any two bodies contacting each other produce a CF, *F*_*c*_ at the immersion site (Figure 4b). *F*_*c*_ can be decomposed into two orthogonal components, normal and parallel to the contact surface. The parallel force is neglected due to extremely low friction coefficient [40]. As a result *F*_*c*_ is reduced to:

(4) Fc=0δ<0kδp−δ˙Chδ,δcδ≥0,

where *δ* is the penetration depth as shown in Figure 4c,d, *k* is the contact stiffness coefficient of the immersion site, *p* is a coefficient defined by the materials’ force deformation properties, and *C* is the damping coefficient. *h* is a sigmoid like step function with a unity gain and a transition interval *δ*_*c*_. In this study, *F*_c_ is computed for cartilage‐to‐cartilage contact with *k* = 500 N/mm, *p* = 1.5, *δ*_*c*_ = 0.01 mm, and *C* = 5 kg/s [9, 42–44].

#### 2.3.3. Initial Condition and Static Equilibrium

In order to calculate force generated by a ligament as in Equation (1), we need the slack lengths *L*_0_ for each ligament, as used in Equation (2). The knee full‐extension position is required for the calculation of the zero‐load ligament lengths. However, since NK MRI were not acquired in full extension, the resulting CM had to be brought to full‐extension to estimate *L*_*e*_, which is necessary to compute *L*_0_ for all the ligaments. After computing *L*_0_, the model is relaxed from its full‐extension posture to an equilibrium state. At steady state, forces are balanced and the joint does not move, the model approaches to its starting NK posture.

#### 2.3.4. Kinematic Validation

In this study, we compared five motions of NK and CM joints during passive flexion motion. Anatomically, NK possesses all six DoF. Flexion–extension is exerted onto the knee joint externally, resulting in passive knee motion. During passive flexion, the knee joint continuously reorients and repositions, resulting in a coupling movement. The remaining knee rotations and translations become dependent on the flexion–extension motion [45]. Therefore, by exerting the same flexion–extension rotation on both NK and CM joints we expect the production of the same angular and linear trajectories. In other words, the remaining dependent motions of the six DoF—abduction–adduction (π/2 − *β*), internal–external (*γ*) rotation, medial–lateral (*S*_1_), anterior–posterior (*S*_2_), and inferior–superior (*S*_3_) translations (Figure 2)—are expected to be similar if CM is adequately constructed.

**Figure 5:**
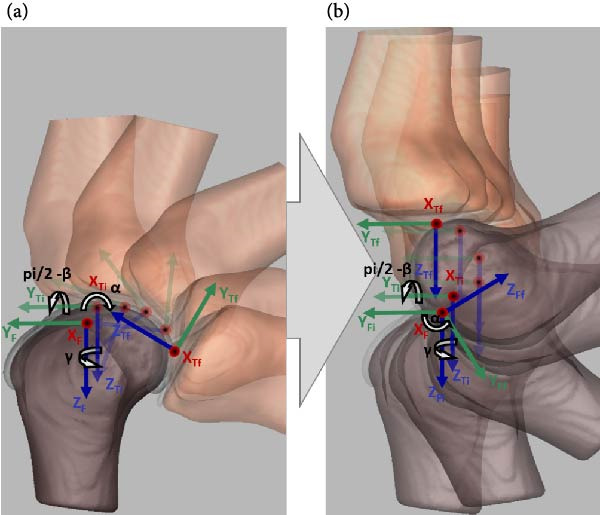
Transformation from (a) tibia moving relative to a fixed femur (FF‐MT) to (b) tibia moving on a flexing femur (HF‐PMT). (a) Tibia moves with respect to the femoral CS fixed to the GCS. (b) Joint flexion–extension is applied to the femur, while the remaining five dependent motions occur on the tibia.

In order to validate the proposed multibody tibiofemoral model, the five dependent motions obtained from NK and computed from CM are compared quantitatively. In the NK model, the tibia moves on a FF as in Figure 5a. With the femur fixed to the global CS (GCS) and the tibia free to move, the motion is termed “FF‐moving tibia” (FF‐MT), where the tibia transitions from *O*_tibi_ = (*X*_Ti_, *Y*_Ti_, *Z*_Ti_) to *O*_tibf_ = (*X*_Tf_, *Y*_Tf_, *Z*_Tf_) via three rotations and translations.

To align with previous studies, the femur’s flexion–extension movement was constrained by a revolute joint at the femoral origin, attached to the GCS (Figure 5b) [16]. Mechanically, the tibia is connected to the GCS with a perpendicular joint located at the tibial origin. Hence, this complex collective set of motions is called “hinged femur‐perpendicular MT” (HF‐PMT) for convenience. As a result, origins of the femoral and GCS frames and their *X*‐axes are common. Consequently, angular displacement in the sagittal plane is femoral flexion–extension rotation about the *X*_F_‐axis, while the other two dependent rotations and three dependent translations are observed on the tibia with respect to the GCS (*X*_Ti_,*Y*_Ti_,*Z*_Ti_ ⇒ *X*_Tf_,*Y*_Tf_,*Z*_Tf_).

#### 2.3.5. Parametric Modeling

In the experimental study, passive knee motion was exerted manually [13, 14]. During PKF, the remaining five motions—internal–external, varum–valgum, medial–lateral, anterior–posterior, and superior–inferior—are coupled with the flexion movement [45]. Therefore, all these dependent movements can be parameterized with respect to flexion–extension rotation. In NK, the knee was flexed to a maximum flexion starting from the full‐extension position and then extended back to near the starting position, forming a full passive knee movement cycle. The repository contains four such cycles consecutively. The same motion parameterization is established in CM for the same four cycles. This establishes NK as the gold standard for validating proposed CM.

#### 2.3.6. Quantitative Comparison NK vs. CM

For each of the five movements described in the section above, CM and NK kinematics are pairwise compared using linear regression analysis, based on:

(5) y=b0+b1x,

where *y* represents the experimental data and *x* represents the simulated data. The model calculates linear relationship parameters: *b*_0_ (intercept), *b*_1_ (slope), root mean squared error (RMSE), fitting quality (adjusted *R* ^2^), and statistical significance for estimation (*p*‐value). If the experimental outputs are similar to the simulated kinematics the regression parameters are expected to be close to one for slope and *R* ^2^, close to zero for RMSE and intercept, and less than 0.05 for *p*‐value.

## 3. Results

Figure 6 presents the five dependent motions from PKF, as detailed in the Method’s Kinematic Validation subsection, for NK experimental and CM simulated motions. Each motion in NK includes four consecutive recordings, with the averages and variances plotted. Flexion–extension data from NK drives CM simulations, generating corresponding trajectories for the five motions. The six graphs in Figure 7 are linear regression model plots for comparing the similarity of the experimental NK and simulated CM trajectories.

Figure 6Pairwise comparison of NK (red) and CM (blue) kinematics. Angular displacements: (a) flexion–extension (input motion), (b) abduction–adduction, and (c) internal–external rotations. Linear displacements: (d) medial–lateral, (e) anterior–posterior, and (f) superior–inferior translations. Subpart (a) includes flexion–extension for completeness, where a straight line is expected.

**Figure:**
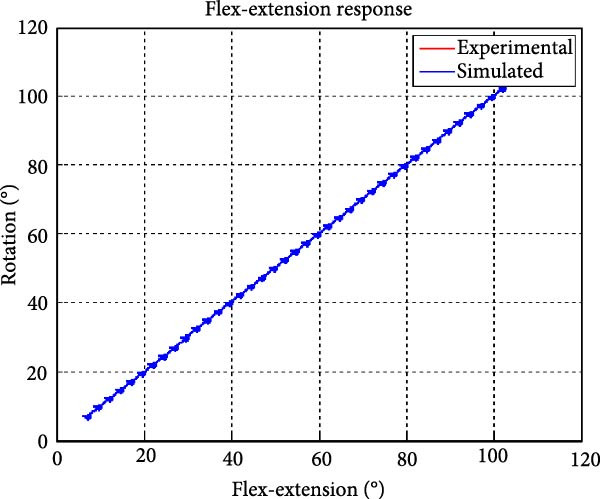
(a)

**Figure:**
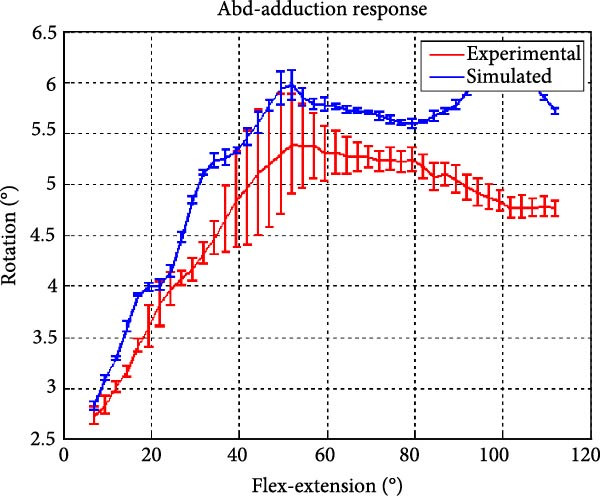
(b)

**Figure:**
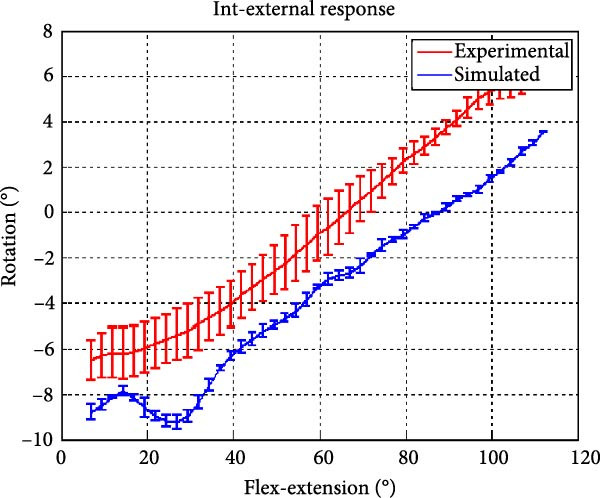
(c)

**Figure:**
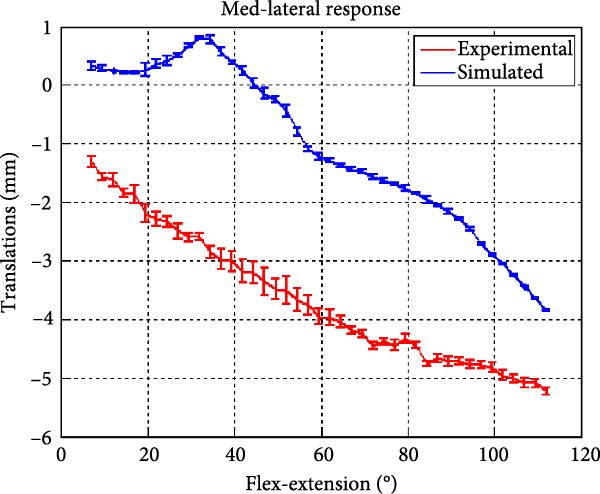
(d)

**Figure:**
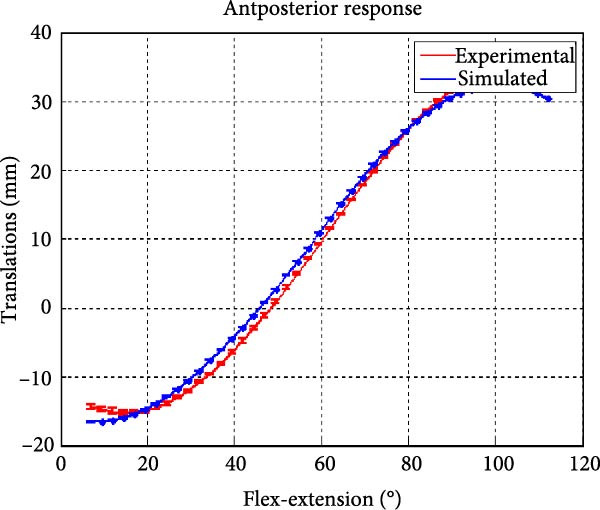
(e)

**Figure:**
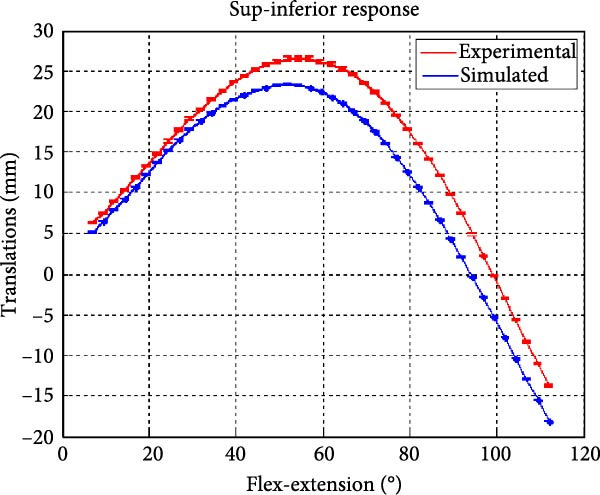
(f)

Figure 7Linear regression plots comparing experimental NK and simulated CM outcomes for PKF. Angular kinematics: (a) flexion–extension, (b) abduction–adduction, and (c) external–internal rotations. Linear kinematics: (d) medial–lateral, (e) anterior–posterior, and (f) superior–inferior translations.

**Figure:**
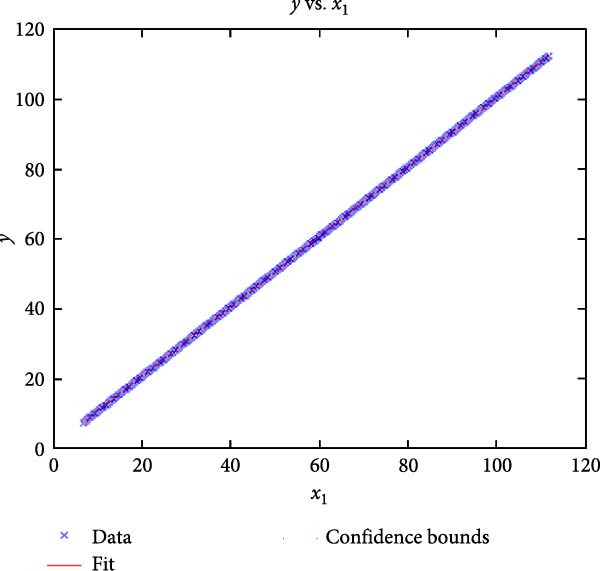
(a)

**Figure:**
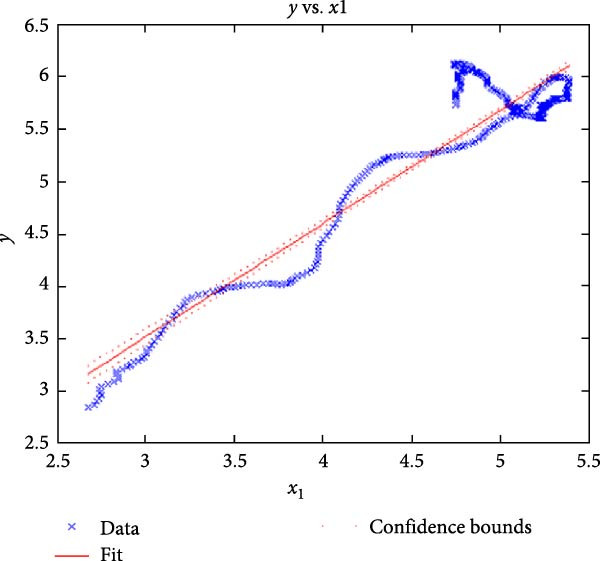
(b)

**Figure:**
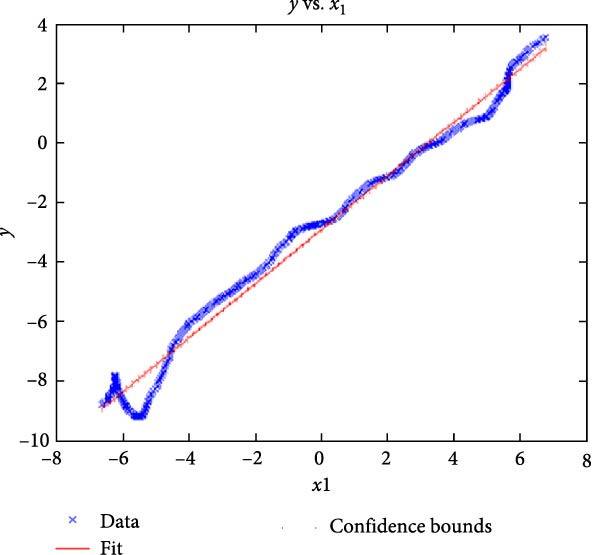
(c)

**Figure:**
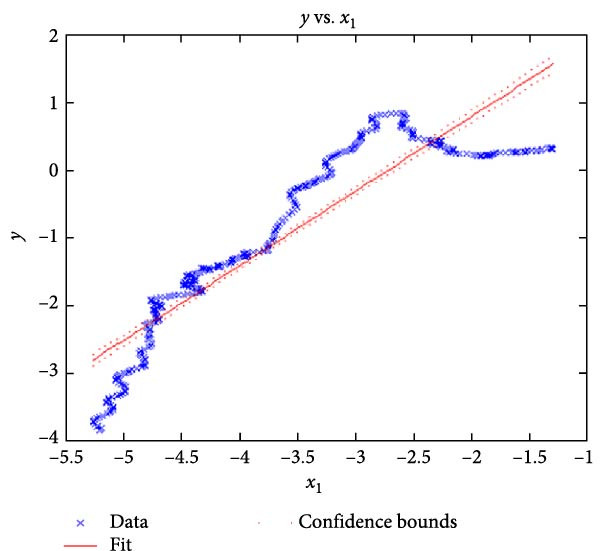
(d)

**Figure:**
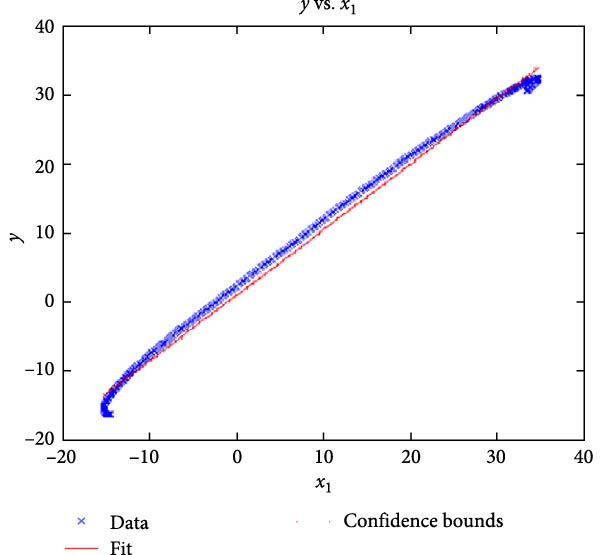
(e)

**Figure:**
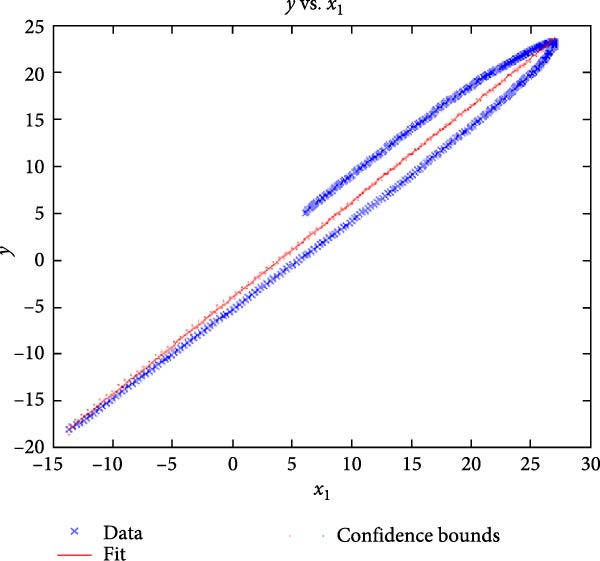
(f)

Finally, Table 1 comprises the results of two ranges of motion; (a) truncated (7°–90°) and (b) higher flexion range (7°–112°) flexion–extension movements. The regression analysis indicate experimental (NK) and simulated (CM) trajectories are highly correlated and the results are statistically significant, with a *p*‐value less than 0.05 for all paired kinematics.

**Table 1:** Linear regression analysis on similarity of NK and CM.

JMs	Slope		Intercept		RMSE		*R* ^2^	
	90°	112°	90°	112°	90°	112°	90°	112°
FE	1	1	0	0	0	0	1	1
AA	1.048	1.084	0.276	0.243	0.148	0.302	0.971	0.875
EI	0.934	0.905	−2.862	−2.973	0.522	0.510	0.971	0.983
ML	0.866	1.104	2.340	2.990	0.439	0.540	0.797	0.839
AP	0.992	0.950	0.862	0.653	1.230	1.420	0.994	0.994
SI	0.889	1.023	−1.215	−4.157	1.780	1.850	0.907	0.973

*Note*: Analysis is performed for the flexion ranges 7–90 and 7–112. Flexion–extension serves as the independent variable and input motion for PKF movement. The remaining DOF represent components of the passive flexion movement, where FE, AA, and IE are rotations, and ML, AP, and SI are translations.

Abbreviations: AA, abduction–adduction; AP, anterior–posterior; DOF, degrees of freedom; FE, flexion–extension; IE, internal–external; JMs, joint motions; ML, medial–lateral; SI, superior–inferior.

Figure 6a shows flexion–extension rotation with respect to itself. It is illustrated for completeness of the argument for all the six knee JMs. This independent motion is linearly sampled and drives the rest of the five dependent JMs for both NK and CM experiments.

Figure 6b depicts the trajectory for abduction–adduction of the knee joint with respect to the flexion–extension rotation for both NK (red) and CM (blue) for the four repetition. The abduction–adduction trajectory resulting from four executions shows noticeable variation between 40° and 60° of flexion–extension. Although it is not as high as in NK, abduction–adduction in CM also varies more noticeably between 40° and 60° of flexion–extension. While average trajectories are not exactly superimposed for NK and CM, the two trajectories are highly correlated as can be seen in Figure 7b and Table 1. Regression results for frontal plane rotation (abduction–adduction) are better in the 7°–90° range than in the 7°–112° ranges, except for the intercept.

The third angular displacement, internal–external rotation, has a close‐to‐linear relation with flexion–extension. While CM internal–external rotation varies less across experiments, as shown in Figure 6c (blue), the NK internal–external curve (Figure 6c, red) is smoother. NK and CM internal–external rotations are highly correlated, as shown in Figure 7c. The external–internal rotation similarity comparison parameters, RMSE, and *R* ^2^ values are slightly better for 7°–112°, whereas slope and intercept values are closer to ideal for 7°–90° of flexion–extension.

The medial–lateral displacement is inversely proportional to flexion–extension. The medial–lateral displacement has the least excursion of all translational displacements. The experimental and simulated medial–lateral displacements do not superimpose, however, their offset is less than 2 mm throughout the experiments. CM medial–lateral displacement (Figure 6d, blue) shows less variance across the entire motion compared to NK (Figure 6d, red) with a wider variation in the range of 40°–60° of flexion–extension. Correlation between NK and CM, although not as high as other dependent motions, is still significant, as shown in Figure 7d and Table 1. For mediolateral translation, slope and *R* ^2^ parameters for 7°–112°, and intercept and RMSE parameters for 7°–90° are marginally better.

Forward and backward movement of the tibia along the floating axis *F* (anterior–posterior displacement) is almost identical for NK (red) and CM (blue), as shown in Figure 6e. The similarity throughout the entire range of flexion–extension between NK and CM is highest for anterior–posterior displacement, as illustrated in Figure 7e, producing the highest correlation in Table 1. The anterior–posterior translation similarity calculations resulted in an *R* ^2^ value of 0.994 for both 7°–90° and 7°–112° of flexion–extension. For 7°–90°, the slope and RMSE parameters are closer to ideal, one. The intercept is better for 7°–112° of flexion–extension.

Figure 6f illustrates mean trajectories of superior–inferior displacements for measured NK (red) and compiled CM (blue) experiments during PKF. Although there are some discrepancies at higher flexions, the resulting curvatures are closely related, producing significant correlation results as shown in Figure 7f and Table 1. When comparing NK superior–inferior translations with the results of CM, the slope and *R* ^2^ values are better for 7–112°, while intercept and RMSE values are better for 7°–90° of flexion–extension.

LF–flexion angle relationships during four consecutive passive knee motion are presented in Figure 8. The anterior bundle of ACL (red) is under tensile stress around 20°–90° of flexion, whereas the posterior bundle, pACL (blue) exhibit highest load at full extension, decreasing gradually and vanishing at around 50° (Figure 8a). The PCL showed progressively increasing forces with knee flexion, with the posterior bundle, pPCL, carrying the majority of the load (Figure 8b). The LCL was primarily loaded near full extension, while the ALS demonstrated increasing stress with greater flexion. MCL components displayed sharing load, with anterior fibers (aMCL), oblique fibers (oMCLa and oMCLp), and posterior bundle of dMCL showing tension at lower flexion angles, whereas anterior bundle of dMCL, dMCLa, become engaged at higher flexion angles (Figure 8d). The joint capsule and popliteal ligaments, although not displayed in the figure, are also under stress at full extension.

Figure 8CM ligaments stress outcomes during four consecutive passive knee motion: (a) anterior (red) and posterior (blue) bundles of ACL, (b) anterior (red) and posterior (blue) bundles of PCL, (c) LCL (red) and ALS (blue), (d) anterior (red) MCL, anterior (blue), and posterior (purple) bundles of deep MCL and anterior (black) and posterior (green) bundles of oblique MCL.

**Figure:**
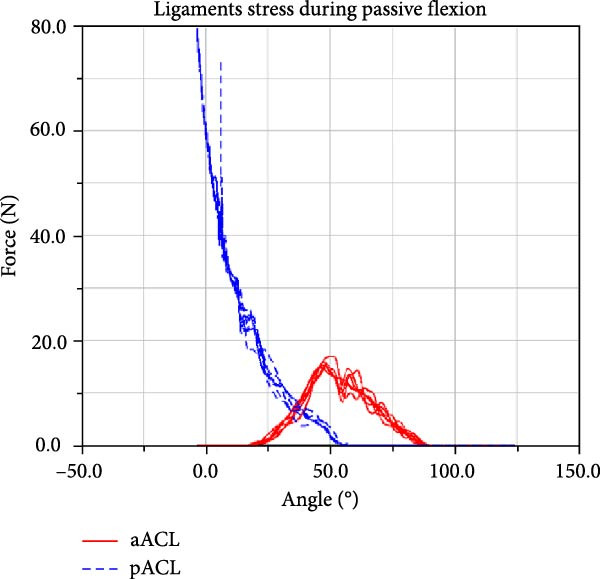
(a)

**Figure:**
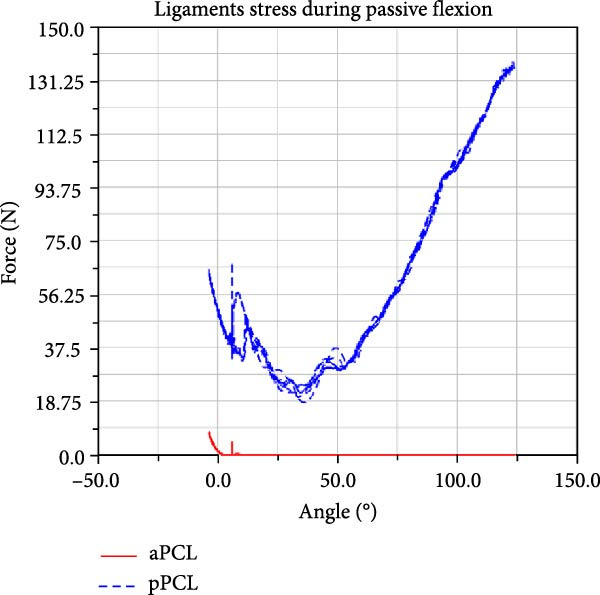
(b)

**Figure:**
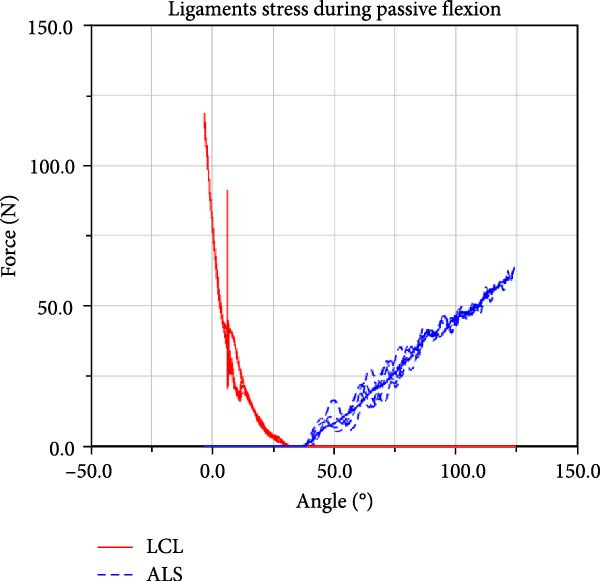
(c)

**Figure:**
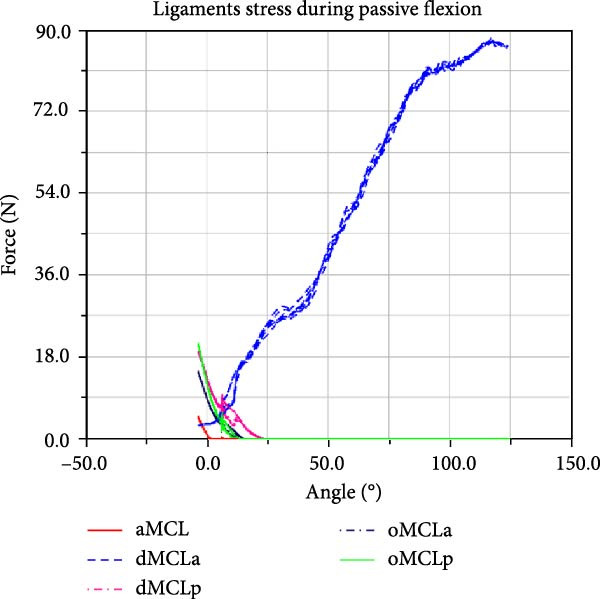
(d)

## 4. Discussion

In conclusion, the simulated model produces a similar response to the actual knee when compared under the generally accepted conditions of passive flexion. In the proposed CM, a multibody tibiofemoral joint is constructed incorporating subject‐specific anatomical structures. Bone and cartilage are segmented from patient MRI as solid shell structures to represent rigid components and contact surfaces. Ligament insertion sites are also defined from MRI, based on anatomical relevancies. This approach allows us to customize the proposed model based on the parameters acquired from the knee joint MR images of the patient. While repository provided CT images offer superior contrast and spatial resolution for bony structures [46], MRI was used exclusively in this study because it provides sufficient accuracy and is clinically preferred as a single imaging modality capable of capturing both soft and hard tissues without exposing patients to ionizing radiation. The joint trajectories produced by the CM are compared with those measured from NK dataset and are discussed in detail in following paragraphs.

A visual comparison in Figure 6 shows that the joint trajectories produced in NK and CM are similar for the same flexion–extension for the input motion. The CM response is within an acceptable range when individual joint parameters are compared pairwise for NK and CM, as illustrated in the Figure 6. This comparison approach aligns with the method used in previous studies in the literature to demonstrate model validity [6, 7, 9]. Additionally, we presented a quantitative method to compare actual and CM responses where the results are shown in Table 1. Pearson’s correlation results between the responses show a strong correlation (*r* > 0.89) for each of the five dependent motions. In this study, a linear regression model of experimental kinematics is fitted to the simulated kinematics (Figure 7). In summary, regression results, RMSE and *R* ^2^ show that the experimental (NK) and simulated (CM) motions are acceptably similar.

LF and CF were not experimentally recorded, however the force outputs of the CM (Figure 8) are consistent with responses reported in the literature [6, 47]. Additionally, anteriorly linear and internal–external angular loading are applied to the CM, results corroborate findings reported in previous study [48]. The model predicted load patterns provide profiles of ligament stress throughout the passive flexion, offering quantitative insight not obtainable in vivo.

The stress behaviors of combined anterior and PCL bundles are in line with reported studies by Kiapour et al. [47] and Ren et al. [48]. They represented connective structures as nonlinear elastic tensile materials defined by cross sectional area, except ACL, PCL, sMCL, and LCL which modeled as FE. Finite element–based knee models provide highly detailed stress and strain analyses but typically demand extensive preprocessing, specialized expertise, and long computational times. In contrast, our multibody framework achieves subject‐specific ligament routing and passive kinematics with markedly lower computational cost and more straightforward implementation. As a result of this efficiency, we expect improved scalability for patient‐specific applications in the future studies, increasing translational potential for surgical planning.

In this study a 3D model platform of the tibiofemoral joint is established. Although patella and meniscus do not contribute significantly to the passive knee motion they will become important when other activities are investigated such as gait, running, and landing. Additionally, cruciate ligaments specifically will be considered as finite element model via segmentation of intact side to extensively analyze loading of the ligaments as in previous studies [47, 48]. The proposed model’s capacity extends to simulating knee pathologies, such as ligament rupture and cartilage degeneration, allowing for the evaluation of surgical modifications in a quantitative manner, assessing regression or progression. Surgeons accustomed to traditional methods, that is, the Drawer test, for ligament assessment and treatment evaluation, could benefit from in performing such tests in simulation environment using the presented tibiofemoral model.

We demonstrated that using MR images of lower leg and knee is sufficient for the reconstruction of an acceptable computational tibiofemoral model customized to a patient. With this approach, although it may increase preparation time, surgeons will have the opportunity to study their approach prior to operation in detail, accounting for several possible outcomes.

## Conflicts of Interest

The authors declare no conflicts of interest.

## Funding

No funding are provided to the study.

## Data Availability

The data are available as Digital Commons@DU (University of Denver) (https://digitalcommons.du.edu/natural_knee_data/).
